# Dynamics of Space-Fractional Euler–Bernoulli and Timoshenko Beams

**DOI:** 10.3390/ma14081817

**Published:** 2021-04-07

**Authors:** Paulina Stempin, Wojciech Sumelka

**Affiliations:** Institute of Structural Analysis, Poznan University of Technology, Piotrowo 5 Street, 60-965 Poznan, Poland; paulina.s.stempin@doctorate.put.poznan.pl

**Keywords:** free vibration, non-local model, fractional calculus, beam

## Abstract

This paper investigates the dynamics of the beam-like structures whose response manifests a strong scale effect. The space-Fractional Euler–Bernoulli beam (s-FEBB) and space-Fractional Timoshenko beam (s-FTB) models, which are suitable for small-scale slender beams and small-scale thick beams, respectively, have been extended to a dynamic case. The study provides appropriate governing equations, numerical approximation, detailed analysis of free vibration, and experimental validation. The parametric study presents the influence of non-locality parameters on the frequencies and shape of modes delivering a depth insight into a dynamic response of small scale beams. The comparison of the s-FEBB and s-FTB models determines the applicability limit of s-FEBB and indicates that the model (also the classical one) without shear effect and rotational inertia can only be applied to beams significantly slender than in a static case. Furthermore, the validation has confirmed that the fractional beam model exhibits very good agreement with the experimental results existing in the literature—for both the static and the dynamic cases. Moreover, it has been proven that for fractional beams it is possible to establish constant parameters of non-locality related to the material and its microstructure, independent of beam geometry, the boundary conditions, and the type of analysis (with or without inertial forces).

## 1. Introduction

In this paper, we concentrate on purely mechanical processes. Within this class of physical problems, one can distinguish two special cases: the first, the so-called dynamic processes, and the second, the so-called static processes. Herein, experiments show that each mechanical process needs time, where time is understood as a non-spatial continuum in which events occur in succession from the past through the present to the future [1]. By analogy, from the theoretical perspective, statics is a special case of dynamics by assuming inertia forces equal zero [2,3]. Therefore, one can conclude that, both from the experimental and the theoretical point of views, the first class is more reliable and enables deeper insight into the characteristic features of analyzed material/structure/mechanism.

The dynamics of the mechanical process (processes in which the inertia forces cannot be neglected) can be analyzed under different time and spatial scales. Depending on the chosen scales, specific experimental techniques and theoretical modeling tools should be applied. From this perspective, it is important to emphasize that as mathematical modeling has crucial meaning in the proceeding sections, and the considered events occur in MHz and sizes of nm are taken into account, the continuum type (phenomenological) modeling (CTM) application is justified. It is characteristic that CTM methodology needs special treatment when the analyzed sizes are of nm order, namely, the scale effect should be included—the reason for such methodology is due to the fact that at such scale the influence intrinsic structure become dominant. Herein, from many concepts, i.e., strain-gradient theories [4,5,6,7], peridynamics [8,9], micropolar theories [10,11,12], integral-type theories [13], general non-local theories [14,15], and theories of material surfaces/surface elasticity theory [16], the one which bases on the fractional calculus (FC) application is used hereinafter [8,17,18] (cf. the review in [19]). The latter concept, hereinafter referred to as space-fractional continuum mechanic (s-FCM), bases on the FC property that derivatives in this formalism being non-local, and therefore the scale effect is included.

Within the class of material bodies, whose dynamics can be analyzed, a special place exists of so-called beams (seminal works of Leonhard Euler and Daniel Bernoulli dates back to 1750). The beams are a special case of general 3D material bodies where two spatial dimensions (beam cross section dimensions) are smaller than the third one (beam length). However, this basic geometric beam definition, from the theoretical point of view, can be more specific depending on proportions of those dimensions, as well as cross section properties (full, thin-walled, open, etc.), e.g., the Euler–Bernoulli beam theory, Timoshenko beam theory, Vlasov beam theory, Reddy–Bickford beam theory [20,21,22], or theories including the axial tensile/compressive force due to either the residual (surface) stresses or the electrostatic (magnetostatic) pulling [23,24]. It is important that many of those classical theories are generalized to include the scale effect. Let us mention here the works by Ghaffari et al. [25], Sumelka et al. [26], Challamel [27], Huang et al. [28], Ramezan et al. [29], Hassanpour and Heppler [30], Zhang et al. [31], or Patnaik et al. [32,33]. However, recently, an original version of the space-Fractional Euler–Bernoulli beam (s-FEBB) [34] and the space-Fractional Timoshenko beam (s-FTB) [35] was formulated in the framework of the general s-FCM [18,36] and moreover identified and validated in nano- and microbeam experimental bending tests.

Based on the above discussion, this paper further develops s-FEBB [26,34] and s-FTB [35] models to the dynamic case. Special attention is focused on the following aspects which manifest the originality of the presented investigation:

- formulation of the general governing equations describing the dynamic behavior of the s-FEBB and s-FTB models;
- elaboration of the numerical algorithms for both fractional beams for the case of free vibrations;
- in-depth study of the influence of non-locality parameters on the eigenfrequencies and the shape of modes;
- determination of the geometric criterion (which is more restrictive than in the statics) according to which s-FEBB can be reasonably applied, in both fractional and classical approaches; and
- identification of fractional beam parameters and validation in the static beam bending and the resonance tests, based on experimental data available in [37,38].

The paper is structured as follows. Section 2 deals with the formulation of the dynamic governing equations for the s-FEBB and s-FTB beams. Section 3 is devoted to the elaboration of the numerical scheme for free vibrations. Section 4 provides a parametric study. Section 5 is related to the experimental validation, and finally Section 6 concludes the paper.

## 2. Governing Equations

The s-FEBB [26,34] and the s-FTB models [35] are formulated by putting proper restrictions on the general space-Fractional Continuum Mechanics (s-FCM) [18,36]. Thus, in the study of transverse vibrations, the displacement field is assumed as follows:

(1) $$\left\{\begin{array}{c} u_{1}\left(x_{1},x_{2},x_{3},t\right)=x_{3}\Phi_{2}\left(x_{1},t\right),\\ u_{2}\left(x_{1},x_{2},x_{3},t\right)=0,\\ u_{3}\left(x_{1},x_{3},x_{3},t\right)={\bar{u}}_{3}\left(x_{1},t\right), \end{array}\right.$$

where $u_{i}$ are the components of the displacement vector, $x_{i}$ are spatial coordinates ($x_{1}$—along the beam, and $x_{2}$ and $x_{3}$ define the plane of the cross section), *t* is time, ${\bar{u}}_{3}\left(x_{1},t\right)$ is the rigid body translation of the cross section in 3rd axis direction at time *t*, and $\Phi_{2}\left(x_{1},t\right)$ is the rigid rotation of cross section (positive keeping the right-hand rule) at time *t*. Let us mention that to make the equations more readable, the following shortcut is additionally assumed throughout the paper:

(2) $$\underset{⏜}{(\;\;)\equiv \left(\;\;\right)\left(x_{1},t\right)},$$

for example, $\underset{⏜}{{\bar{u}}_{3}}={\bar{u}}_{3}\left(x_{1},t\right)$.

Rotation $\underset{⏜}{\Phi_{2}}$ is defined as the Riesz–Caputo fractional derivative of ${\bar{u}}_{3}$ with the proportionality factor $\ell_{f}^{\alpha -1}$ and for s-FTB is extended by an additional rotation due to the fractional shear deformation ${\overset{⋄}{\gamma }}_{13}$,

(3) $$\underset{⏜}{\Phi_{2}}=\left\{\begin{array}{c} \begin{array}{cc} -\ell_{f}^{\alpha -1}\overset{\alpha }{\underset{x_{1}}{D}}\underset{⏜}{{\bar{u}}_{3}}\;\;\;\;\; & \mathrm{for}\;\text{s-FEBB},\\ -\ell_{f}^{\alpha -1}\overset{\alpha }{\underset{x_{1}}{D}}\underset{⏜}{{\bar{u}}_{3}}+\underset{⏜}{{\overset{⋄}{\gamma }}_{13}}\; & \mathrm{for}\;\text{s-FTB}, \end{array} \end{array}\right.$$

where

(4) $$\overset{\alpha }{\underset{x}{D}}f\left(x\right){=}_{x-\ell_{f}}^{RC}D_{x+\ell_{f}}^{\alpha }f\left(x\right)=\frac{1}{2}\frac{\Gamma (2-\alpha )}{\Gamma (2)}\left({\;}_{x-\ell_{f}}^{C}D_{x}^{\alpha }f\left(x\right)+{\left(-1\right)}^{n}{\;}_{x}^{C}D_{x+\ell_{f}}^{\alpha }f\left(x\right)\right),$$

with the left-side and right-side Caputo derivatives

(5) $${\;}_{x-\ell_{f}}^{C}D_{x}^{\alpha }f\left(x\right)=\frac{1}{\Gamma (n-\alpha )}\int_{x-\ell_{f}}^{x}\frac{f^{\left(n\right)}\left(\tau \right)}{{\left(x-\tau \right)}^{\alpha -n+1}}d\tau ,$$

(6) $${\;}_{x}^{C}D_{x+\ell_{f}}^{\alpha }f\left(x\right)=\frac{-1}{\Gamma (n-\alpha )}\int_{x}^{x+\ell_{f}}\frac{f^{\left(n\right)}\left(\tau \right)}{{\left(x-\tau \right)}^{\alpha -n+1}}d\tau ,$$

where Γ is the Euler gamma function; n=[α]+1; [·] denotes the integer part of a real number; α∈(0,1〉 is the order of derivative; and $\ell_{f}$ is the length scale, i.e., the surrounding affecting the considered material point. The concept of variable length scale $\ell_{f}=\ell_{f}\left(x\right)$, as function decreasing at the boundaries, has been kept [39]. These two parameters—α and $\ell_{f}$—are regarded as being associated with microstructure [40] and responsible for scale effect mapping.

Next, based on the s-FCM formalism, bending moment $\underset{⏜}{M_{2}}$, shear force $\underset{⏜}{V_{3}}$, and distributed dynamic load $\underset{⏜}{p_{3}}$ take the following form (for the derivations, see Appendix A):

(7) $$\underset{⏜}{M_{2}}=\left\{\begin{array}{c} \begin{array}{cc} -\ell_{f}^{\alpha -1}\overset{\alpha }{\underset{x_{1}}{D}}\left(\ell_{f}^{\alpha -1}\overset{\alpha }{\underset{x_{1}}{D}}\underset{⏜}{{\bar{u}}_{3}}\right)EI\;\;\; & \mathrm{for}\;\text{s-FEBB},\\ \ell_{f}^{\alpha -1}\left[-\overset{\alpha }{\underset{x_{1}}{D}}\left(\ell_{f}^{\alpha -1}\overset{\alpha }{\underset{x_{1}}{D}}\underset{⏜}{{\bar{u}}_{3}}\right)+\overset{\alpha }{\underset{x_{1}}{D}}\underset{⏜}{{\overset{⋄}{\gamma }}_{13}}\right]EI\; & \mathrm{for}\;\text{s-FTB}, \end{array} \end{array}\right.$$

(8) $$\underset{⏜}{V_{3}}=kGA\underset{⏜}{{\overset{⋄}{\gamma }}_{13}}\;\mathrm{for}\;s-\mathrm{FTB},$$

(9) $$\underset{⏜}{V_{3}}=\left\{\begin{array}{c} \begin{array}{cc} \overset{\alpha }{\underset{x_{1}}{D}}\left(\underset{⏜}{M_{2}}\ell_{f}^{\alpha -1}\right)\;\;\;\;\; & \mathrm{for}\;\text{s-FEBB},\\ \overset{\alpha }{\underset{x_{1}}{D}}\left(\underset{⏜}{M_{2}}\ell_{f}^{\alpha -1}\right)-\rho I\underset{⏜}{{\ddot{\Phi }}_{2}}\; & \mathrm{for}\;\text{s-FTB}, \end{array} \end{array}\right.$$

(10) $$\begin{array}{c} \underset{⏜}{p_{3}}=-\overset{\alpha }{\underset{x_{1}}{D}}\left(\underset{⏜}{V_{3}}\ell_{f}^{\alpha -1}\right)+\rho A\underset{⏜}{{\ddot{u}}_{3}}, \end{array}$$

where *E* is the Young’s modulus, $G=\frac{E}{2(1+\nu )}$ is the Kirchhoff modulus, ν is Poisson’s ratio, *k* is the so-called shear correction factor, $I=\int_{A}x_{3}^{2}\;dA$ denotes the moment of inertia, ρ is density, and $\ddot{\left(\;\;\right)}=\frac{d^{2}\left(\;\right)}{dt^{2}}$. By introducing Equations (3) and (7) into Equations (9) and (10), the shear force and the distributed load are determined in terms of ${\bar{u}}_{3}$, ${\overset{⋄}{\gamma }}_{13}$ and their time derivatives, namely,

(11) $$\underset{⏜}{V_{3}}=\left\{\begin{array}{c} \begin{array}{cc} -\overset{\alpha }{\underset{x_{1}}{D}}\left[\ell_{f}^{2\alpha -2}\overset{\alpha }{\underset{x_{1}}{D}}\left(\ell_{f}^{\alpha -1}\overset{\alpha }{\underset{x_{1}}{D}}\underset{⏜}{{\bar{u}}_{3}}\right)\right]EI\;\;\;\;\;\;\;\;\;\;\;\;\;\;\;\; & \mathrm{for}\;\text{s-FEBB},\\ \overset{\alpha }{\underset{x_{1}}{D}}\left\{\ell_{f}^{2\alpha -2}\left[-\overset{\alpha }{\underset{x_{1}}{D}}\left(\ell_{f}^{\alpha -1}\overset{\alpha }{\underset{x_{1}}{D}}\underset{⏜}{{\bar{u}}_{3}}\right)+\overset{\alpha }{\underset{x_{1}}{D}}\underset{⏜}{{\overset{⋄}{\gamma }}_{13}}\right]\right\}EI+\rho I\left(\ell_{f}^{\alpha -1}\overset{\alpha }{\underset{x_{1}}{D}}\underset{⏜}{{\ddot{\bar{u}}}_{3}}-\underset{⏜}{{\ddot{\overset{⋄}{\gamma }}}_{13}}\right)\; & \mathrm{for}\;\text{s-FTB}. \end{array} \end{array}\right.$$

(12) $$\underset{⏜}{p_{3}}=\left\{\begin{array}{c} \begin{array}{cc} \overset{\alpha }{\underset{x_{1}}{D}}\left\{\ell_{f}^{\alpha -1}\overset{\alpha }{\underset{x_{1}}{D}}\left[\ell_{f}^{2\alpha -2}\overset{\alpha }{\underset{x_{1}}{D}}\left(\ell_{f}^{\alpha -1}\overset{\alpha }{\underset{x_{1}}{D}}\underset{⏜}{{\ddot{\bar{u}}}_{3}}\right)\right]\right\}EI+\rho A\underset{⏜}{{\ddot{\bar{u}}}_{3}}\;\;\;\;\;\; & \mathrm{for}\;\text{s-FEBB}\\ \begin{array}{cc} \overset{\alpha }{\underset{x_{1}}{D}} & \left\{\ell_{f}^{\alpha -1}\overset{\alpha }{\underset{x_{1}}{D}}\left[\ell_{f}^{2\alpha -2}\overset{\alpha }{\underset{x_{1}}{D}}\left(\ell_{f}^{\alpha -1}\overset{\alpha }{\underset{x_{1}}{D}}\underset{⏜}{{\ddot{\bar{u}}}_{3}}\right)\right]\right\}EI-\overset{\alpha }{\underset{x_{1}}{D}}\left[\ell_{f}^{\alpha -1}\overset{\alpha }{\underset{x_{1}}{D}}\left(\ell_{f}^{2\alpha -2}\overset{\alpha }{\underset{x_{1}}{D}}\underset{⏜}{{\ddot{\overset{⋄}{\gamma }}}_{13}}\right)\right]EI+\\ \; & -\rho I\overset{\alpha }{\underset{x_{1}}{D}}\left(\ell_{f}^{2\alpha -2}\overset{\alpha }{\underset{x_{1}}{D}}\underset{⏜}{{\ddot{\bar{u}}}_{3}}\right)+\rho I\overset{\alpha }{\underset{x_{1}}{D}}\left(\ell_{f}^{\alpha -1}\underset{⏜}{{\ddot{\overset{⋄}{\gamma }}}_{13}}\right)+\rho A\underset{⏜}{{\ddot{\bar{u}}}_{3}}. \end{array}\; & \mathrm{for}\;\text{s-FTB} \end{array} \end{array}\right.$$

Note that the s-FTB beam requires an additional equation, which was obtained from equating Equation (8) and Equation (11_2_). Finally, the general governing equations of beams under the transversely distributed load $p_{3}\left(x_{1},t\right)$ are

(13) $$\left\{\begin{array}{c} \begin{array}{cc} \overset{\alpha }{\underset{x_{1}}{D}}\left\{\ell_{f}^{\alpha -1}\overset{\alpha }{\underset{x_{1}}{D}}\left[\ell_{f}^{2\alpha -2}\overset{\alpha }{\underset{x_{1}}{D}}\left(\ell_{f}^{\alpha -1}\overset{\alpha }{\underset{x_{1}}{D}}\underset{⏜}{{\bar{u}}_{3}}\right)\right]\right\}EI+\rho A\underset{⏜}{{\ddot{\bar{u}}}_{3}}=\underset{⏜}{p_{3}}\;\;\;\;\;\;\;\; & \mathrm{for}\;\text{s-FEBB}\\ \left\{\begin{array}{c} \begin{array}{cc} \overset{\alpha }{\underset{x_{1}}{D}} & \left\{\ell_{f}^{\alpha -1}\overset{\alpha }{\underset{x_{1}}{D}}\left[\ell_{f}^{2\alpha -2}\overset{\alpha }{\underset{x_{1}}{D}}\left(\ell_{f}^{\alpha -1}\overset{\alpha }{\underset{x_{1}}{D}}\underset{⏜}{{\bar{u}}_{3}}\right)\right]\right\}EI-\overset{\alpha }{\underset{x_{1}}{D}}\left[\ell_{f}^{\alpha -1}\overset{\alpha }{\underset{x_{1}}{D}}\left(\ell_{f}^{2\alpha -2}\overset{\alpha }{\underset{x_{1}}{D}}\underset{⏜}{{\overset{⋄}{\gamma }}_{13}}\right)\right]EI+\\ \; & -\rho I\overset{\alpha }{\underset{x_{1}}{D}}\left(\ell_{f}^{2\alpha -2}\overset{\alpha }{\underset{x_{1}}{D}}\underset{⏜}{{\ddot{\bar{u}}}_{3}}\right)+\rho A\underset{⏜}{{\ddot{\bar{u}}}_{3}}+\rho I\overset{\alpha }{\underset{x_{1}}{D}}\left(\ell_{f}^{\alpha -1}\underset{⏜}{{\ddot{\overset{⋄}{\gamma }}}_{13}}\right)=\underset{⏜}{p_{3}}, \end{array}\\ \overset{\alpha }{\underset{x_{1}}{D}}\left\{\ell_{f}^{2\alpha -2}\left[\overset{\alpha }{\underset{x_{1}}{D}}\left(\ell_{f}^{\alpha -1}\overset{\alpha }{\underset{x_{1}}{D}}\underset{⏜}{{\bar{u}}_{3}}\right)-\overset{\alpha }{\underset{x_{1}}{D}}\underset{⏜}{{\overset{⋄}{\gamma }}_{13}}\right]\right\}EI-\rho I\left(\ell_{f}^{\alpha -1}\overset{\alpha }{\underset{x_{1}}{D}}\underset{⏜}{{\ddot{\bar{u}}}_{3}}-\underset{⏜}{{\ddot{\overset{⋄}{\gamma }}}_{13}}\right)+kGA\underset{⏜}{{\overset{⋄}{\gamma }}_{13}}=0 \end{array}\right. & \mathrm{for}\;\text{s-FTB} \end{array} \end{array}\right.$$

Note that neglecting of the shear deformation $\underset{⏜}{{\overset{⋄}{\gamma }}_{13}}$ in Equation (3_2_) and the rotary inertia of cross section $\frac{1}{2}\int_{V}\rho x_{3}^{2}\underset{⏜}{{\dot{\Phi }}_{2}^{2}}dV$ in Equation (A10_2_) results in the reduction of the s-FTB model to the s-FEBB model. Moreover, neglecting the inertia force, i.e., the kinetic energy K=0 (Equation (A10)), leads to equations of statics [34,35]

(14) $$\left\{\begin{array}{c} \begin{array}{cc} \overset{\alpha }{\underset{x_{1}}{D}}\left\{\ell_{f}^{\alpha -1}\overset{\alpha }{\underset{x_{1}}{D}}\left[\ell_{f}^{2\alpha -2}\overset{\alpha }{\underset{x_{1}}{D}}\left(\ell_{f}^{\alpha -1}\overset{\alpha }{\underset{x_{1}}{D}}{\bar{u}}_{3}\left(x_{1}\right)\right)\right]\right\}EI=p_{3}\left(x_{1}\right)\;\;\;\;\;\;\;\;\;\; & \mathrm{for}\;\text{s-FEBB}\\ \left\{\begin{array}{c} \begin{array}{cc} \overset{\alpha }{\underset{x_{1}}{D}} & \left\{\ell_{f}^{\alpha -1}\overset{\alpha }{\underset{x_{1}}{D}}\left[\ell_{f}^{2\alpha -2}\overset{\alpha }{\underset{x_{1}}{D}}\left(\ell_{f}^{\alpha -1}\overset{\alpha }{\underset{x_{1}}{D}}{\bar{u}}_{3}\left(x_{1}\right)\right)\right]\right\}EI-\overset{\alpha }{\underset{x_{1}}{D}}\left[\ell_{f}^{\alpha -1}\overset{\alpha }{\underset{x_{1}}{D}}\left(\ell_{f}^{2\alpha -2}\overset{\alpha }{\underset{x_{1}}{D}}{\overset{⋄}{\gamma }}_{13}\left(x_{1}\right)\right)\right]EI=p_{3}\left(x_{1}\right), \end{array}\\ \overset{\alpha }{\underset{x_{1}}{D}}\left\{\ell_{f}^{2\alpha -2}\left[\overset{\alpha }{\underset{x_{1}}{D}}\left(\ell_{f}^{\alpha -1}\overset{\alpha }{\underset{x_{1}}{D}}{\bar{u}}_{3}\left(x_{1}\right)\right)-\overset{\alpha }{\underset{x_{1}}{D}}{\overset{⋄}{\gamma }}_{13}\left(x_{1}\right)\right]\right\}EI+kGA{\overset{⋄}{\gamma }}_{13}\left(x_{1}\right)=0 \end{array}\right. & \mathrm{for}\;\text{s-FTB} \end{array} \end{array}\right.$$

Herein, because in this paper we focus on the analysis of the free vibration, i.e., neglecting the transverse dynamic load ($p_{3}\left(x_{1},t\right)=0$) and assuming the separation of variables, namely,

(15) $$\underset{⏜}{{\bar{u}}_{3}}={\bar{u}}_{3}\left(x_{1},t\right)={\bar{u}}_{3}\left(x_{1}\right)e^{i\omega t},\;\underset{⏜}{{\ddot{\bar{u}}}_{3}}={\ddot{\bar{u}}}_{3}\left(x_{1},t\right)=-\omega^{2}{\bar{u}}_{3}\left(x_{1}\right)e^{i\omega t}.$$

we have

(16) $$\left\{\begin{array}{c} \begin{array}{cc} \overset{\alpha }{\underset{x_{1}}{D}}\left\{\ell_{f}^{\alpha -1}\overset{\alpha }{\underset{x_{1}}{D}}\left[\ell_{f}^{2\alpha -2}\overset{\alpha }{\underset{x_{1}}{D}}\left(\ell_{f}^{\alpha -1}\overset{\alpha }{\underset{x_{1}}{D}}{\bar{u}}_{3}\right)\right]\right\}EI-\rho A\omega^{2}{\bar{u}}_{3}=0\;\;\;\;\;\;\;\; & \mathrm{for}\;\text{s-FEBB}\\ \left\{\begin{array}{c} \begin{array}{cc} \overset{\alpha }{\underset{x_{1}}{D}} & \left\{\ell_{f}^{\alpha -1}\overset{\alpha }{\underset{x_{1}}{D}}\left[\ell_{f}^{2\alpha -2}\overset{\alpha }{\underset{x_{1}}{D}}\left(\ell_{f}^{\alpha -1}\overset{\alpha }{\underset{x_{1}}{D}}{\bar{u}}_{3}\right)\right]\right\}EI-\overset{\alpha }{\underset{x_{1}}{D}}\left[\ell_{f}^{\alpha -1}\overset{\alpha }{\underset{x_{1}}{D}}\left(\ell_{f}^{2\alpha -2}\overset{\alpha }{\underset{x_{1}}{D}}{\overset{⋄}{\gamma }}_{13}\right)\right]EI+\\ \; & +\rho I\omega^{2}\overset{\alpha }{\underset{x_{1}}{D}}\left(\ell_{f}^{2\alpha -2}\overset{\alpha }{\underset{x_{1}}{D}}{\bar{u}}_{3}\right)-\rho A\omega^{2}{\bar{u}}_{3}-\rho I\omega^{2}\overset{\alpha }{\underset{x_{1}}{D}}\left(\ell_{f}^{\alpha -1}{\overset{⋄}{\gamma }}_{13}\right)=0, \end{array}\\ \overset{\alpha }{\underset{x_{1}}{D}}\left\{\ell_{f}^{2\alpha -2}\left[\overset{\alpha }{\underset{x_{1}}{D}}\left(\ell_{f}^{\alpha -1}\overset{\alpha }{\underset{x_{1}}{D}}{\bar{u}}_{3}\right)-\overset{\alpha }{\underset{x_{1}}{D}}{\overset{⋄}{\gamma }}_{13}\right]\right\}EI+kGA\overset{⋄}{\gamma }+\rho I\omega^{2}\left(\ell_{f}^{\alpha -1}\overset{\alpha }{\underset{x_{1}}{D}}{\bar{u}}_{3}-{\overset{⋄}{\gamma }}_{13}\right)=0, \end{array}\right. & \mathrm{for}\;\text{s-FTB} \end{array} \end{array}\right.$$

where ${\bar{u}}_{3}={\bar{u}}_{3}\left(x_{1}\right)$ and ${\overset{⋄}{\gamma }}_{13}={\overset{⋄}{\gamma }}_{13}\left(x_{1}\right)$.

As a concluding remark of this Section, let us mention that it is characteristic for the fractional formulation that taking α=1 leads directly to the classical formulation, i.e., Equation (16) reduces to the classical Euler–Bernoulli beam (CEBB) and the classical Timoshenko beam (CTB) theories, respectively,

(17) $$\left\{\begin{array}{c} \begin{array}{cc} \frac{d^{4}{\bar{u}}_{3}}{dx_{1}^{4}}EI-\rho A\omega^{2}{\bar{u}}_{3}=0\;\;\;\;\;\;\;\;\; & \mathrm{for}\;\mathrm{CEBB},\\ \left\{\begin{array}{c} \frac{d^{4}{\bar{u}}_{3}}{dx_{1}^{4}}EI-\frac{d^{3}\gamma_{13}}{dx_{1}^{3}}EI+\rho I\omega^{2}\frac{d^{2}{\bar{u}}_{3}}{dx_{1}^{2}}-\rho A\omega^{2}{\bar{u}}_{3}-\rho I\omega^{2}\frac{d\gamma_{13}}{dx_{1}}=0,\\ \frac{d^{3}{\bar{u}}_{3}}{dx_{1}^{3}}EI-\frac{d^{2}\gamma_{13}}{dx_{1}^{2}}EI+\rho I\omega^{2}\left(\frac{d{\bar{u}}_{3}}{dx_{1}}-\gamma_{13}\right)+kGA\gamma_{13}=0 \end{array}\right. & \mathrm{for}\;\mathrm{CTB}. \end{array} \end{array}\right.$$

## 3. Numerical Study

**Figure 1:**
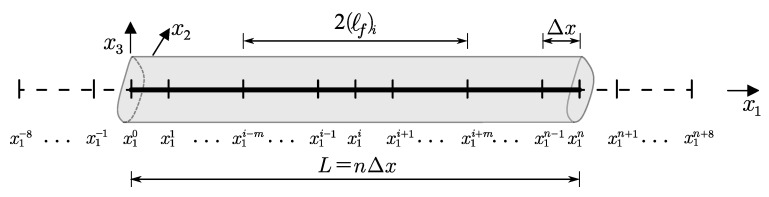
Discretization of the analyzed beam of length *L*—homogeneous grid: real nodes $x_{1}^{0}÷x_{1}^{n}$, fictitious nodes $x_{1}^{-8}÷x_{1}^{-1}$, and $x_{1}^{n+1}÷x_{1}^{n+8}$.

A discrete model approximates the elaborated beam governing equations Equation (16) by analogy to the work in [41]. Therefore, the beam is divided into *n* intervals of length Δx by assigning real nodes $x_{1}^{0}÷x_{1}^{n}$ (see Figure 1), whereas the Caputo fractional derivatives $\overset{\alpha }{\underset{x_{1}}{D}}\left(\;.\;\right)$ are approximated by the trapezoidal rule together with the finite difference method [41,42,43]), which results in additional fictitious nodes ($x_{1}^{-8}÷x_{1}^{-1}$ and $x_{1}^{n+1}÷x_{1}^{n+8}$) outside the beam. The elimination of these points is discussed at the end of this Section.

Based on the above numerical scheme, the discrete version of the Equation (16) is given as follows (for numerical representation of shear force Equation (11): bending moment Equation (7) and rotation Equation (3), see Appendix B).

(18) $$\left\{\begin{array}{c} \begin{array}{cc} D_{i}EI-\rho A\omega^{2}{\bar{u}}_{3}\left(x_{1}^{i}\right)=0\;\;\;\;\;\;\;\;\; & \mathrm{for}\;\text{s-FEBB},\\ \left\{\begin{array}{c} D_{i}EI-C_{i}EI+\rho I\omega^{2}B_{i}^{\left\{2\right\}}-\rho A\omega^{2}{\bar{u}}_{3}\left(x_{1}^{i}\right)-\rho I\omega^{2}A_{i}^{\left\{2\right\}}=0\\ C_{i}EI-B_{i}EI+kGA{\overset{⋄}{\gamma }}_{13}\left(x_{1}^{i}\right)+\rho I\omega^{2}\left({\left(\ell_{f}^{\alpha -1}\right)}_{i}A_{i}-{\overset{⋄}{\gamma }}_{13}\left(x_{1}^{i}\right)\right)=0 \end{array}\right. & \mathrm{for}\;\text{s-FTB}, \end{array} \end{array}\right.$$

where

(19) $$D_{i}=\overset{\alpha }{\underset{x_{1}}{D}}\left\{{\left(\ell_{f}^{\alpha -1}\right)}_{i}\overset{\alpha }{\underset{x_{1}}{D}}\left[{\left(\ell_{f}^{2\alpha -2}\right)}_{i}\overset{\alpha }{\underset{x_{1}}{D}}\left({\left(\ell_{f}^{\alpha -1}\right)}_{i}\overset{\alpha }{\underset{x_{1}}{D}}{\bar{u}}_{3}\left(x_{1}^{i}\right)\right)\right]\right\}=\overset{\alpha }{\underset{x_{1}}{D}}\left[{\left(\ell_{f}^{\alpha -1}C\right)}_{i}\right],$$

(20) $$C_{i}=\overset{\alpha }{\underset{x_{1}}{D}}\left[{\left(\ell_{f}^{2\alpha -2}\right)}_{i}\overset{\alpha }{\underset{x_{1}}{D}}\left({\left(\ell_{f}^{\alpha -1}\right)}_{i}\overset{\alpha }{\underset{x_{1}}{D}}{\bar{u}}_{3}\left(x_{1}^{i}\right)\right)\right]=\overset{\alpha }{\underset{x_{1}}{D}}\left[{\left(\ell_{f}^{2\alpha -2}B\right)}_{i}\right],$$

(21) $$B_{i}=\overset{\alpha }{\underset{x_{1}}{D}}\left[{\left(\ell_{f}^{\alpha -1}\right)}_{i}\overset{\alpha }{\underset{x_{1}}{D}}{\bar{u}}_{3}\left(x_{1}^{i}\right)\right]=\overset{\alpha }{\underset{x_{1}}{D}}\left[{\left(\ell_{f}^{\alpha -1}A\right)}_{i}\right],\;\;B_{i}^{\left\{2\right\}}=\overset{\alpha }{\underset{x_{1}}{D}}\left[{\left(\ell_{f}^{2\alpha -2}\right)}_{i}\overset{\alpha }{\underset{x_{1}}{D}}{\bar{u}}_{3}\left(x_{1}^{i}\right)\right]=\overset{\alpha }{\underset{x_{1}}{D}}\left[{\left(\ell_{f}^{2\alpha -2}A\right)}_{i}\right],$$

(22) $$A_{i}=\overset{\alpha }{\underset{x_{1}}{D}}{\bar{u}}_{3}\left(x_{1}^{i}\right),$$

(23) $$C_{i}=\overset{\alpha }{\underset{x_{1}}{D}}\left[{\left(\ell_{f}^{\alpha -1}\right)}_{i}\overset{\alpha }{\underset{x_{1}}{D}}\left({\left(\ell_{f}^{2\alpha -2}\right)}_{i}\overset{\alpha }{\underset{x_{1}}{D}}{\overset{⋄}{\gamma }}_{13}\left(x_{1}^{i}\right)\right)\right]=\overset{\alpha }{\underset{x_{1}}{D}}\left[{\left(\ell_{f}^{\alpha -1}B\right)}_{i}\right],$$

(24) $$B_{i}=\overset{\alpha }{\underset{x_{1}}{D}}\left[{\left(\ell_{f}^{2\alpha -2}\right)}_{i}\overset{\alpha }{\underset{x_{1}}{D}}{\overset{⋄}{\gamma }}_{13}\left(x_{1}^{i}\right)\right]=\overset{\alpha }{\underset{x_{1}}{D}}\left[{\left(\ell_{f}^{2\alpha -2}A\right)}_{i}\right],$$

(25) $$A_{i}=\overset{\alpha }{\underset{x_{1}}{D}}{\overset{⋄}{\gamma }}_{13}\left(x_{1}^{i}\right),\;\;A_{i}^{\left\{2\right\}}=\overset{\alpha }{\underset{x_{1}}{D}}\left[{\left(\ell_{f}^{\alpha -1}\right)}_{i}{\overset{⋄}{\gamma }}_{13}\left(x_{1}^{i}\right)\right],$$

where

(26) $$\overset{\alpha }{\underset{x_{1}}{D}}{\left(\;.\;\right)}_{i}=h^{1-\alpha }A\left[B{\left(\;.\;\right)}_{i-m}^{\prime }+\sum_{j^{a}=i-m+1}^{i-1}C^{a}{\left(\;.\;\right)}_{j^{a}}^{\prime }+2{\left(\;.\;\right)}_{i}^{\prime }+\sum_{j^{b}=i+1}^{i+m-1}C^{b}{\left(\;.\;\right)}_{j^{b}}^{\prime }+B{\left(\;.\;\right)}_{i+m}^{\prime }\right],$$

and

(27) $$\begin{array}{c} h=\Delta x,\;m=m_{i}={\left(\ell_{f}\right)}_{i}/h\geq 2,\;A=\frac{\Gamma (2-\alpha )}{2\Gamma (2)\Gamma (3-\alpha )},\\ B={\left(m-1\right)}^{2-\alpha }-\left(m+\alpha -2\right)m^{1-\alpha },\\ C^{a}={\left(i-j^{a}+1\right)}^{2-\alpha }-2{\left(i-j^{a}\right)}^{2-\alpha }+{\left(i-j^{a}-1\right)}^{2-\alpha },\\ C^{b}={\left(j^{b}-i+1\right)}^{2-\alpha }-2{\left(j^{b}-i\right)}^{2-\alpha }+{\left(j^{b}-i-1\right)}^{2-\alpha }. \end{array}$$

The forward, backward, or central difference scheme is applied in Equation (19) ÷ (25) to approximate the first derivatives ${\left(\;.\;\right)}_{i}^{\prime }$ at point *i* in accordance with Equation (28) and Table 1,

(28) $${\left(\;.\;\right)}_{i}^{\prime }=\left[-{\left(\;.\;\right)}_{i+N_{1}-N_{2}}+{\left(\;.\;\right)}_{i+N_{1}+N_{2}}\right]\frac{1}{2N_{2}h}.$$

It is also clear that, as in Equation (16), applying α=1 in Equation (18) causes a return for $N_{1}=0$ and $N_{2}=\frac{1}{2}$ to the classical central difference scheme ($A=\frac{1}{2}$, $B=C^{a}=C^{b}=0$); for details, please see Appendix B.

As mentioned, the application of the variable length scale $\ell_{f}\left(x\right)$, which is decreasing at the boundaries [39], results in only eight fictitious nodes ($x_{1}^{-8}÷x_{1}^{-1},$$x_{1}^{n+1}÷x_{1}^{n+8}$) on each side of the beam. These points are eliminated, by the analogy to the approach presented in [39], according to the following procedure.

- By equating of the central and forward finite difference schemes at points $x_{1}^{-6}÷x_{1}^{-1}$ and the central and backward finite difference schemes at points $x_{1}^{n+1}÷x_{1}^{n+6}$ for the fourth-order derivative of displacement,
  (29) $$\begin{array}{c} -u_{i-2}+4u_{i-1}-5u_{i}+5u_{i+2}-4u_{i+3}+u_{i+4}=0,\;\mathrm{for}\;i=-6÷-1,\\ -u_{i+2}+4u_{i+1}-5u_{i}+5u_{i-2}-4u_{i-3}+u_{i-4}=0\;\mathrm{for}\;i=n+1÷n+6; \end{array}$$
- additionally for s-FTB model, by equating the central and forward finite difference schemes at points $x_{1}^{-4}÷x_{1}^{1}$ and the central and backward finite difference schemes at points $x_{1}^{n-1}÷x_{1}^{n+4}$ for the third order derivative of strain,
  (30) $$\begin{array}{c} -{\overset{⋄}{\gamma }}_{i-2}+2{\overset{⋄}{\gamma }}_{i-1}+2{\overset{⋄}{\gamma }}_{i}-8{\overset{⋄}{\gamma }}_{i+1}+7{\overset{⋄}{\gamma }}_{i+2}-2{\overset{⋄}{\gamma }}_{i+3}=0,\;\mathrm{for}\;i=-4÷1,\\ -{\overset{⋄}{\gamma }}_{i+2}+2{\overset{⋄}{\gamma }}_{i+1}+2{\overset{⋄}{\gamma }}_{i}-8{\overset{⋄}{\gamma }}_{i-1}+7{\overset{⋄}{\gamma }}_{i-2}-2{\overset{⋄}{\gamma }}_{i-3}=0,\;\mathrm{for}\;i=n-1÷n+4 \end{array}$$

Finally, the system of equations has to be completed by the boundary conditions. Table 2 and Table 3 indicate how the boundary conditions for selected static schemes are included in the algorithm of the s-FEBB and s-FTB models, respectively.

## 4. Parametric Study

This section focuses on the comprehensive free vibration analysis of the non-local s-FEBB and s-FTB beams. The considered beams are geometrically determined by the length *L*, width *a*, and height *b* of the rectangular cross section with the shear correction factor k=5/6, and are made of the material described by the density ρ, Young’s modulus *E*, and Poisson’s ratio ν. The study focused especially on two static schemes—a fixed beam and a cantilever beam. In all examples, Δx=0.001L is assumed. The results are presented in a non-dimensional form, i.e., as the eigenvectors normalized by displacement and the dimensionless frequency $\bar{f}$, where the relation between $\bar{f}$ and frequency $f=\frac{\omega }{2\pi }$ is

(31) $$f=\bar{f}\frac{\pi^{2}}{L^{2}}\sqrt{\frac{EI}{\rho A}}.$$

The dimensionless frequency $\bar{f}$ is independent on the material and beam geometry as regards s-FEBB, and in the case of s-FTB, it depends only on the length to height L/b ratio and Poisson’s ratio. Therefore, knowing $\bar{f}$ lets one easily calculate by Equation (31) the frequency *f* for beam of arbitrary size and made of arbitrary material.

**Figure 2:**
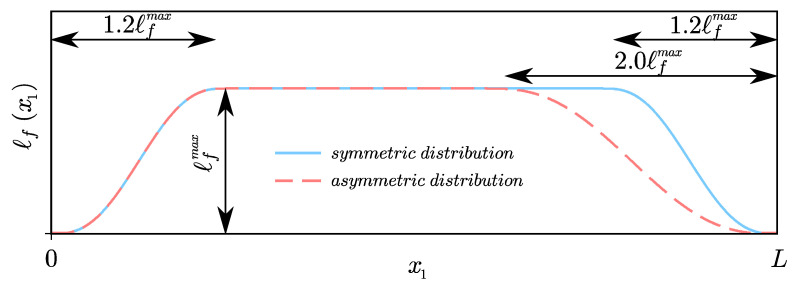
Smooth symmetric and asymmetric distribution of the length scale $\ell_{f}$ along the beam length.

**Figure 3:**
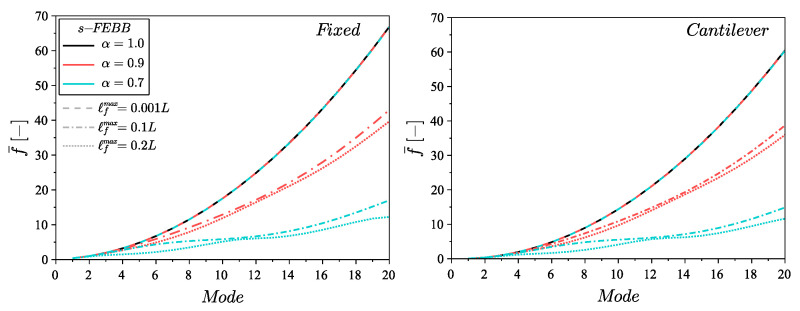
Dimensionless frequency $\bar{f}$ for the s-FEBB model for the fixed beam and cantilever beam for α=[0.9,0.7] and $\ell_{f}^{max}=\left[0.001L,\;0.1L,\;0.2L\right]$ compared to the results of CEBB model (s-FEBB model with α=1.0).

**Figure 4:**
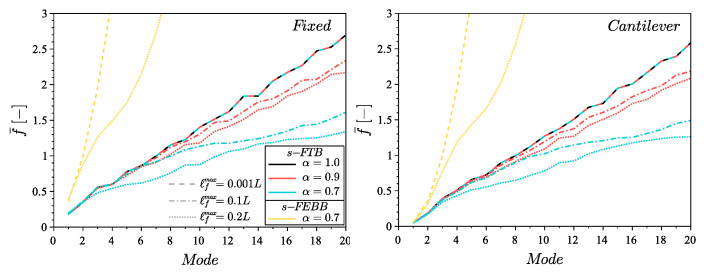
Dimensionless frequency $\bar{f}$ for the s-FTB model for the fixed beam and cantilever beam with length to height ratio L/b=2 and Poisson’s ratio ν=0.2, for α=[0.9,0.7] and $\ell_{f}^{max}=\left[0.001L,\;0.1L,\;0.2L\right]$ compared to the results of CTB model (s-FTB model with α=1.0) and the results of s-FEBB model with α=0.7.

To indicate the effect of the non-locality parameters on the eigenfrequencies and the eigenmodes, the analysis for the s-FTB and s-FEBB models was performed for the following parameters: α=[0.7,0.9] and $\ell_{f}^{max}=\left[0.001L,\;0.1L,\;0.2L\right]$ with the smooth symmetric distribution (see Figure 2). The results of the classical approach (i.e., for α=1.0) are also provided. Figure 3 presents the dimensionless frequency $\bar{f}$ for the s-FEBB model depending on α and $\ell_{f}^{max}$ parameters and Figure 4 for the s-FTB model with L/b = 2 and Poisson’s ratio ν=0.2. It can be observed that the frequencies decrease as the α decreases or as the $\ell_{f}^{max}$ increases. This is especially noticeable for higher modes—for example, for the 20th mode in the case of the fixed beam, $\bar{f}$ decreases from 66.86 for α=1.0 to 12.22 for α=0.7, $\ell_{f}^{max}=0.2L$. Only the first two frequencies of the fixed beam exhibit different behavior—the frequency increases for the 1st mode and for the 2nd mode with $\ell_{f}^{max}=0.1L$, and decreases for the 2nd mode with $\ell_{f}^{max}=0.2L$.

**Figure 5:**
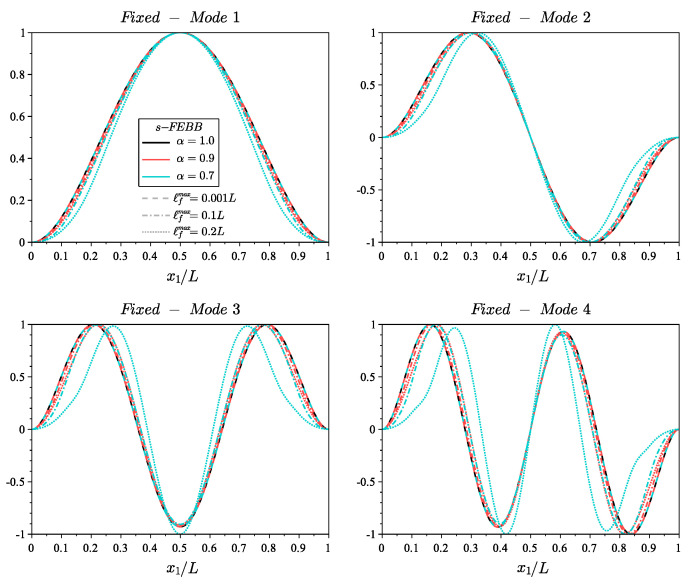
Shape of first four modes of fixed beam for s-FEBB model with α=[0.9,0.7] and $\ell_{f}^{max}=\left[0.001L,\;0.1L,\;0.2L\right]$ compared to the results of CEBB model (s-FEBB with α=1.0).

**Figure 6:**
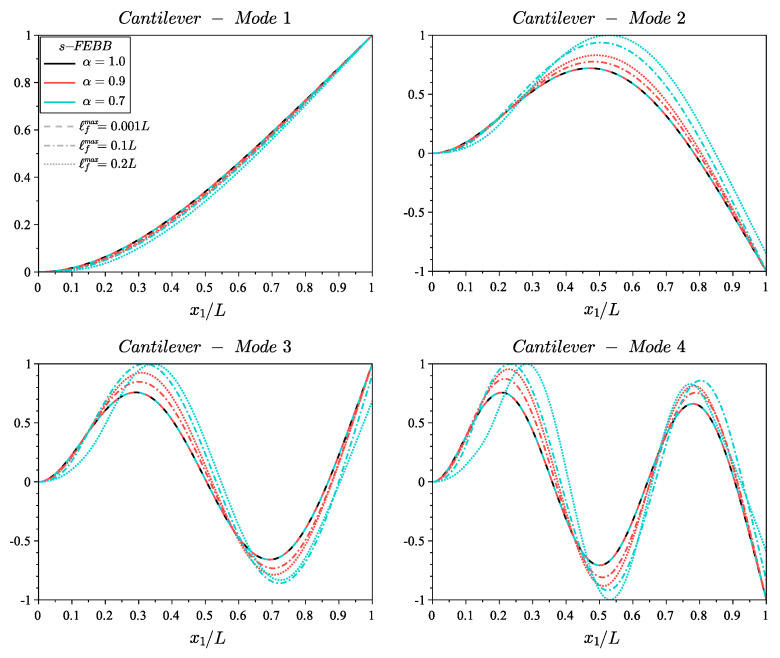
Shape of first four modes of cantilever beam for s-FEBB model with α=[0.9,0.7] and $\ell_{f}^{max}=\left[0.001L,\;0.1L,\;0.2L\right]$ compared to the results of CEBB model (s-FEBB with α=1.0).

**Figure 7:**
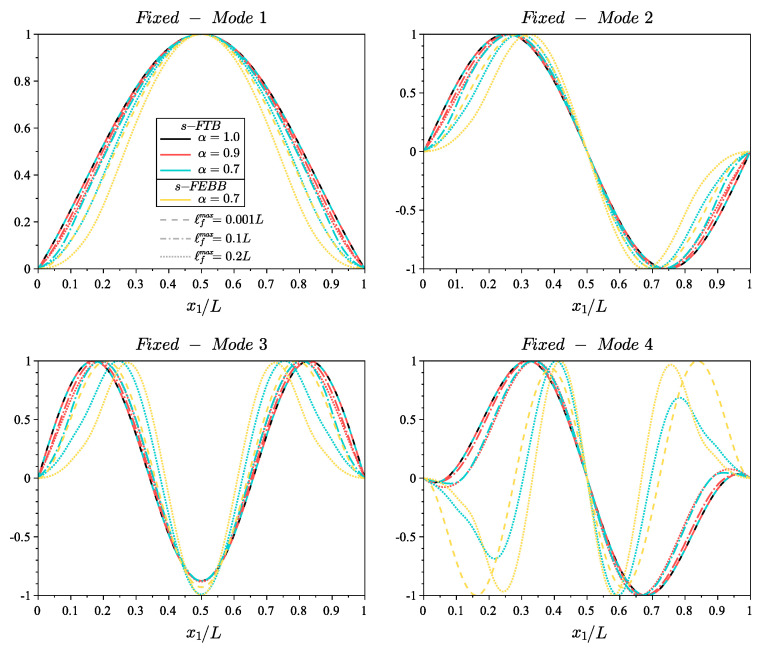
Shape of first four modes of fixed beam with length to height ratio L/b=2 and Poisson’s ratio ν=0.2 for s-FTB model with α=[0.9,0.7] and $\ell_{f}^{max}=\left[0.001L,\;0.1L,\;0.2L\right]$ compared to the results of CTB model (s-FTB with α=1.0) and the results of s-FEBB model with α=0.7.

**Figure 8:**
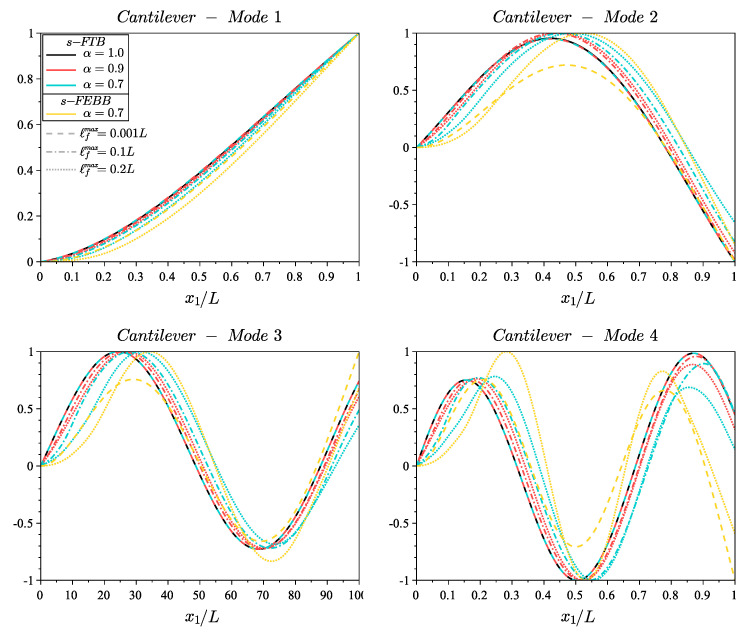
Shape of first four modes of cantilever beam with length to height ratio L/b=2 and Poisson’s ratio ν=0.2 for s-FTB model with α=[0.9,0.7] and $\ell_{f}^{max}=\left[0.001L,\;0.1L,\;0.2L\right]$ compared to the results of CTB model (s-FTB with α=1.0) and the results of s-FEBB model with α=0.7.

Figure 5 and Figure 6 show the shapes of the first four modes for fixed and cantilever beams, respectively, according to the s-FEBB model, while Figure 7 and Figure 8 according to the s-FTB model with L/b=2 and Poisson’s ratio ν=0.2. It should be highlighted that with parameters α and $\ell_{f}$, it is possible to control not only the frequency value, but also the shape of modes. Note that when $\ell_{f}$ is small in relation to *L* ($\ell_{f}^{max}=0.001L$), a local solution—identical to the classic formulation—is obtained.

**Figure 9:**
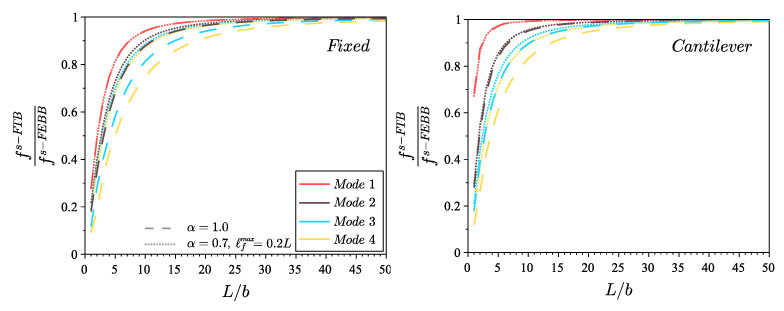
Comparison of the frequency value of first four modes for s-FTB and s-FEBB models for the fixed scheme and the cantilever scheme for α=0.7 and $\ell_{f}^{max}=0.2L$, contrasted with the results for CTB and CEBB models (s-FTB and s-FEBB models with α=1.0).

In addition, note that the results differ considerably for beams with small L/b ratio—both the frequency value (see Figure 4) and the shape of the eigenmodes (see Figure 7 and Figure 8)—when accounting for the shear effect and the rotational inertia. Therefore, Figure 9 shows a comparison of the results obtained according to the s-FTB and s-FEBB models for beams with slenderness (L/b) in the range of 1÷50 to highlight when s-FTB can be successfully simplified to s-FEBB. Additionally, Table 4 presents the geometric criterion L/b of the beam for which a difference between in frequencies received in the Timoshenko model and the Euler–Bernoulli model is less than 5%, for both the classical (α=1.0) and fractional (α=0.7, $\ell_{f}^{max}=0.2L$) approaches. This difference is more significant for the higher mode numbers, both in the local and non-local theories. In the case of dynamic analysis, the CTB can be reduced to the CEBB with the same beam geometry limitation as for static, only for the first eigenvalue. For higher frequencies, the CEBB can be used reasonably and without loss of correctness only for significantly slender beams. However, taking into account the non-locality effect means that the s-FTB can be reduced to the s-FEBB for beams with less slenderness compared to the classical beams.

## 5. Experimental Validation

In models defined within the framework of space-fractional mechanics, the non-locality parameters α and $\ell_{f}$ are considered to be related to the microstructure of the material (for a discussion on the association of microstructure with the non-locality parameters see in [40]). Consequently, these parameters are considered as constant and independent of the body geometry or the analysis performed (static or dynamic). This feature of the fractional models is an advantage over competitive non-local theories in which the length scale is a non-material parameter and depends on dimensions [44,45] and analysis type [46,47]. Regarding the above interpretation, we have conducted a validation in the case of statics (see Equation (14)) and dynamics (see Equations (16) and (18)), and identified new material parameters, α and $\ell_{f}$, for gallium nitride (GaN) nanowires in addition to Young’s modulus E=295GPa and density $\rho =6150\;{\mathrm{kg}/m}^{3}$.

**Figure 10:**
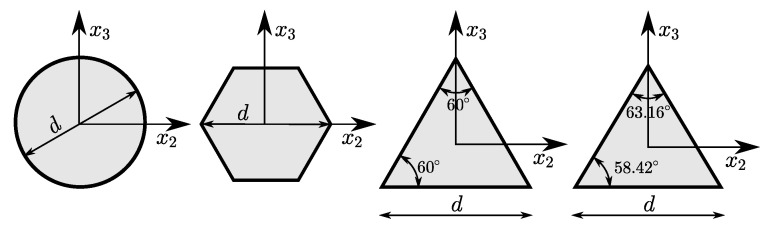
Circular and hexagonal cross sections used in bending test, and triangular (equilateral and isosceles) cross sections used in resonance test.

The static case is related to the bending test [37]. The nanowires with length *L* and circular or hexagonal cross section with diameter *d* (see Figure 10) were loaded at or near the midpoint (at a non-dimensional distance $L_{1}$) with the load *P*. The data of bending beams are collected in a Table 5. The static scheme is fixed beam for case (a) and simply supported beam for cases (b–d). The L/d ratio is greater than 23, therefore, the theory of s-FEBB (see Equation (14_1_)) was used to predict the deflections of considered nanobeams.

**Table 5:** Beam dimensions, load position, and material parameters for GaN nanobeams in bending test [37] (see also Figure 11).

	Cross- Section	Diameter *d* [*nm*]	Length *L* [*nm*]	Load *P* [*nN*]	*L*_1_ [−]	Elastic Modulus *E* [*GPa*]	*α* [−]	$\ell_{f}^{\max}$ [*nm*]
(a)	circle	57.0	3054	122.7	0.54	295	0.66	160
(b)	haxagon	89.3	2398	112.3	0.55			
(c)		97.8	2465	127.7	0.60			
(d)		109.7	2558	200.0	0.53			

However, the dynamic case is related to the resonance analysis of cantilever beams with length *L* and triangular (equilateral or isosceles) cross section with a base length *d* (see Figure 10). The oscillations of nanobeams were excited in the $x_{3}$ direction with the resonance frequency $f_{x_{3}}^{EXP}$ and in the $x_{2}$ direction with the resonance frequency $f_{x_{2}}^{EXP}$ (see Figure 12). The data of beams from the resonance test are collected in a Table 6. We included uncertainties in dimensions measurement (±2 nm in *d* and ±50 nm in *L*) given in [38] and uncertainties in frequency measurement (±3%). The L/d ratio is greater than 63 for all beams tested; therefore, the theory of s-FEBB (see Figure 9; Equations (16)_1_ and (18)_1_) was used to predict the frequencies of considered nanobeams.

**Figure 11:**
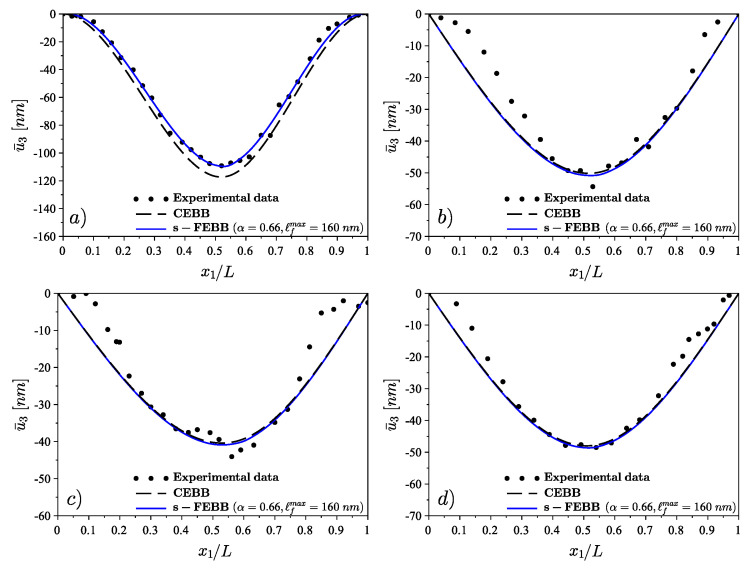
The comparison of experimental measurements [37] (deflections) of GaN nanobeams vs. the classical model (CEBB) and s-FEBB model: (**a**) fixed scheme and (**b**–**d**) simply supported scheme, for load position depending on data in Table 5 (see also Equation (14_1_)).

**Figure 12:**
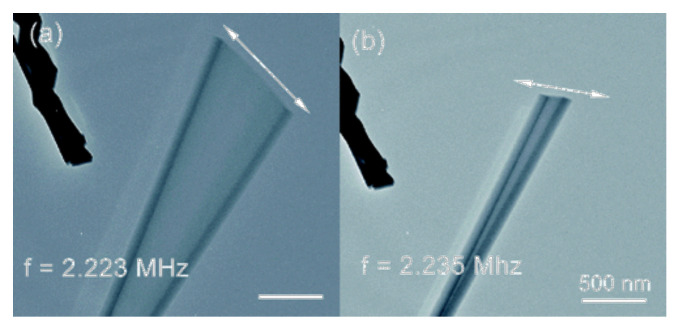
GaN nanowire in resonance, (**a**) in the $x_{3}$ direction and (**b**) in the $x_{2}$ direction, reprinted with permission from C.-Y. Nam, P. Jaroenapibal, D. Tham, D. E. Luzzi, S. Evoy, J. E. Fischer, *Diameter dependent electromechanical properties of GaN nanowires*, Nano Letters 6 (2006) 153–158 [38] Copyright (2006) American Chemical Society.

**Figure 13:**
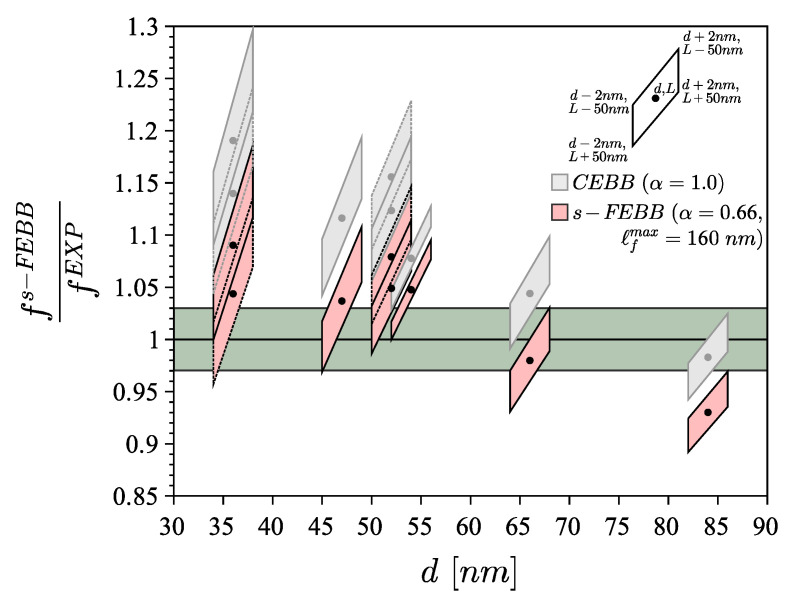
The comparison of experimental measurements [38] (resonance frequencies $f^{EXP}$, see also Table 6) of GaN nanobeams vs. classical model (CEBB) and s-FEBB model (see also Equations (16_1_) and (18_1_)).

For all nanowires analyses mentioned above, we assume Δx=2.398nm. It was found that α=0.66 and $\ell_{f}^{max}=160\;\mathrm{nm}$ with symmetrical distribution in the case of the same boundary conditions (fixed and simply supported beams) at both ends and asymmetrical distribution in case of cantilever beam (see Figure 2). It was established that these parameters are identical for the statics and the dynamics. The results of beam bending analysis (predicted deflections) are presented in Figure 11, while the results of resonance analysis (predicted resonance frequencies to experimental measurements ratio $\frac{f^{s-FEBB}}{f^{EXP}}$) are presented in Figure 13. Concluding, in both statics and dynamics analyses, the analysis results agree very well with the measurement data.

In addition, the results for the CEBB model are also presented to demonstrate that it cannot capture experimental data in both the bending test (see Figure 11) and resonance test (see Figure 13) of small-scale beams.

The presented validation confirms that α and $\ell_{f}^{max}$ are independent of the cross section shape, the cross section dimensions, the length of the beam, the boundary conditions, and the type of analysis, which allows us to deduce that these parameters are being truly material-dependent.

## 6. Conclusions

In this study, the s-FEBB and s-FTB theories were extended to the case of dynamics, and the free vibrations for both models were analyzed. A detailed discussion on the influence of the non-local parameters (α and $\ell_{f}$), as well as the shear effect and the rotational inertia, on the dimensionless frequency and shape of modes leads to the following conclusions:

- the non-local parameters α and $\ell_{f}$ control the frequency value and the shape of a specific mode of the fractional beam;
- taking into account the small-scale effect results in lower frequency values compared to the classical beam theories (except for the first two frequencies of the fixed beam);
- the higher mode number, the more significant the non-locality effect;
- taking into account the shear effect and rotational inertia in the s-FTB results in lower frequency values compared to the s-FEBB;
- in the case of dynamics, the Timoshenko beam model should be considered even for significantly slender beams, which is in contrast to the case of static analysis, especially for the higher mode numbers;
- including the non-locality means that the shear effect and the rotational inertia can be neglected for beams with a smaller length to height L/b ratio compared to the local approach;
- the higher mode number, the more significant is the difference in frequencies received in Timoshenko and Euler–Bernoulli theory, both in the fractional and in the classical approach;
- validation has confirmed that the fractional beam model exhibits very good agreement with the experimental results for both the static and the dynamic cases; and
- it has been proven that for the fractional beams it is possible to establish constant parameters α and $\ell_{f}^{max}$ related to the material and its microstructure, independent of the beam geometry, the boundary conditions, and the type of analysis.

## Figures and Tables

**Table 1:** The applied finite difference schemes in dependence on the position of discretization point *i* (see also Equation (28) and Figure 1).

	Forward	Backward	Central	Central
	$N_{1}=\frac{1}{2},$	$N_{1}=-\frac{1}{2},$	$N_{1}=0,$	$N_{1}=0,$
	$N_{2}=\frac{1}{2}$	$N_{2}=\frac{1}{2}$	$N_{2}=\frac{1}{2}$	$N_{2}=1$
$u_{i}^{\prime }$	i=−8	i=n+8	i=−7.5÷n+7.5	i=−7÷2;n−2÷n+7
${\left(\ell_{f}^{\alpha -1}A\right)}_{i}^{\prime }$	i=−6	i=n+6	i=−5÷n+5	
${\left(\ell_{f}^{2\alpha -2}B\right)}_{i}^{\prime }$	i=−4	i=n+4	i=−3.5÷n+3.5	i=−3÷n+3
${\left(\ell_{f}^{\alpha -1}C\right)}_{i}^{\prime }$	i=−2	i=n+2	i=−1÷n+1	
${\overset{⋄}{\gamma }}_{i}^{\prime }$	i=−6	i=n+6	i=−5.5÷n+5.5	i=−5÷2;n−2÷n+5
${\left(\ell_{f}^{2\alpha -2}A\right)}_{i}^{\prime }$	i=−4	i=n+4	i=−3.0÷n+3.0	
${\left(\ell_{f}^{\alpha -1}B\right)}_{i}^{\prime }$	i=−2	i=n+2		i=−1÷n+1

**Table 2:** Boundary conditions for s-FEBB applied for the selected static schemes (see also  Equations (18), (A12) ÷ (A14), and Figure 1).

Beam Type	Conditions for s-FEBB	
Simply supported	${\bar{u}}_{3}\left(x_{0}\right)=0$, $M_{2}\left(x_{0}\right)=0$,	$u_{0}=0$, $B_{0}=0$
	${\bar{u}}_{3}\left(x_{n}\right)=0$ $M_{2}\left(x_{n}\right)=0$	$u_{n}=0$, $B_{n}=0$
Fixed	${\bar{u}}_{3}\left(x_{0}\right)=0$, $\Phi_{2}\left(x_{0}\right)=0$,	$u_{0}=0$, $A_{0}=0$
	${\bar{u}}_{3}\left(x_{n}\right)=0$ $\Phi_{2}\left(x_{0}\right)=0$	$u_{n}=0$, $A_{n}=0$
Propped cantilever	${\bar{u}}_{3}\left(x_{0}\right)=0$, $\Phi_{2}\left(x_{0}\right)=0$,	$u_{0}=0$, $A_{0}=0$
	${\bar{u}}_{3}\left(x_{n}\right)=0$ $M_{2}\left(x_{n}\right)=0$	$u_{n}=0$, $B_{n}=0$
Cantilever	${\bar{u}}_{3}\left(x_{0}\right)=0$, $\Phi_{2}\left(x_{0}\right)=0$,	$u_{0}=0$, $A_{0}=0$
	$M_{2}\left(x_{n}\right)=0$ $V_{3}\left(x_{n}\right)=0$	$B_{n}=0$, $C_{n}=0$

**Table 3:** Boundary conditions for s-FTB applied for the selected static scheme (see also  Equations (18), (A12) ÷ (A14), and Figure 1).

Beam Type	Conditions for s-FTB	
Simply supported	${\bar{u}}_{3}\left(x_{0}\right)=0$,	$u_{0}=0$,
	$M_{2}\left(x_{0}\right)=0$,	$-B_{0}+A_{0}=0$
	${\bar{u}}_{3}\left(x_{n}\right)=0$	$u_{n}=0$,
	$M_{2}\left(x_{n}\right)=0$	$-B_{n}+A_{n}=0$
Fixed	${\bar{u}}_{3}\left(x_{0}\right)=0$,	$u_{0}=0$,
	$\Phi_{2}\left(x_{0}\right)=0$,	$-{\left(\ell_{f}^{\alpha -1}\right)}_{0}A_{0}+\left(-C_{0}+B_{0}\right)\frac{EI}{kGA}-\frac{\rho I\omega^{2}}{kGA}\left({\left(\ell_{f}^{\alpha -1}\right)}_{0}A_{0}-{\overset{⋄}{\gamma }}_{0}\right)=0$
	${\bar{u}}_{3}\left(x_{n}\right)=0$	$u_{n}=0$,
	$\Phi_{2}\left(x_{n}\right)=0$	$-{\left(\ell_{f}^{\alpha -1}\right)}_{n}A_{n}+\left(-C_{n}+B_{n}\right)\frac{EI}{kGA}-\frac{\rho I\omega^{2}}{kGA}\left({\left(\ell_{f}^{\alpha -1}\right)}_{n}A_{n}-{\overset{⋄}{\gamma }}_{n}\right)=0$
Propped cantilever	${\bar{u}}_{3}\left(x_{0}\right)=0$,	$u_{0}=0$,
	$\Phi_{2}\left(x_{0}\right)=0$,	$-{\left(\ell_{f}^{\alpha -1}\right)}_{0}A_{0}+\left(-C_{0}+B_{0}\right)\frac{EI}{kGA}-\frac{\rho I\omega^{2}}{kGA}\left({\left(\ell_{f}^{\alpha -1}\right)}_{0}A_{0}-{\overset{⋄}{\gamma }}_{0}\right)=0$
	${\bar{u}}_{3}\left(x_{n}\right)=0$	$u_{n}=0$,
	$M_{2}\left(x_{n}\right)=0$	$-B_{n}+A_{n}=0$
Cantilever	${\bar{u}}_{3}\left(x_{0}\right)=0$,	$u_{0}=0$,
	$\Phi_{2}\left(x_{0}\right)=0$,	$-{\left(\ell_{f}^{\alpha -1}\right)}_{0}A_{0}+\left(-C_{0}+B_{0}\right)\frac{EI}{kGA}-\frac{\rho I\omega^{2}}{kGA}\left({\left(\ell_{f}^{\alpha -1}\right)}_{0}A_{0}-{\overset{⋄}{\gamma }}_{0}\right)=0$
	$M_{2}\left(x_{n}\right)=0$	$-B_{n}+A_{n}=0$,
	$V_{3}\left(x_{n}\right)=0$	$\left(-C_{n}+B_{n}\right)EI-\rho I\omega^{2}\left({\left(\ell_{f}^{\alpha -1}\right)}_{n}A_{n}-{\overset{⋄}{\gamma }}_{n}\right)=0$

**Table 4:** Geometric condition L/b of the beam with a difference between the Timoshenko model and the Euler–Bernoulli model less than 5%, for classical (α=1.0) and fractional (α=0.7, $\ell_{f}^{max}=0.2L$) approaches (see also Figure 9).

Beam Type		L/b≥			
		Mode 1	Mode 2	Mode 3	Mode 4
Fixed	Classical (α=1.0)	11	17	22	28
	α=0.7, $\ell_{f}^{max}=0.2L$	11	15	16	17
Cantilever	Classical (α=1.0)	4	10	15	21
	α=0.7, $\ell_{f}^{max}=0.2L$	4	9	13	16

**Table 6:** Beam dimensions, measured frequencies, and material parameters for GaN nanobeams in resonance test [38] (see also Figure 13).

No.	Cross-Section	Diameter d(±2)[nm]	Length L(±50)[nm]	Frequency $f_{x_{3}}^{\mathrm{EXP}}\left(\pm 3\%\right)$ [*MHz*]	Frequency $f_{x_{2}}^{\mathrm{EXP}}\left(\pm 3\%\right)$ [*MHz*]	Elastic Modulus *E* [*GPa*]	Density $\rho \;[\frac{\mathrm{kg}}{m^{3}}]$	*α* [−]	$\ell_{f}^{\max}$ [*nm*]
1	isosceles triangle	36	3200	2.194	2.440	295	6150	0.66	160
2	equilateral triangle	47	3950	2.135	2.135				
3	isosceles triangle	52	4300	1.860	1.925				
4	equilateral triangle	66	4700	2.264	2.264				
5	equilateral triangle	54	11,200	0.316	0.316				
6	equilateral triangle	84	5500	2.223	2.235				

## Data Availability

Data is contained within this article.

## Appendix A

The fractional Cauchy strains are defined as

(A1) $${\overset{⋄}{\varepsilon }}_{ij}=\frac{1}{2}\ell_{f}^{\alpha -1}\left(\overset{\alpha }{\underset{x_{j}}{D}}u_{i}\left(x\right)+\overset{\alpha }{\underset{x_{i}}{D}}u_{j}\left(x\right)\right),$$

therefore the only nonzero components are

(A2) $${\overset{⋄}{\varepsilon }}_{11}={\overset{⋄}{\varepsilon }}_{11}\left(x_{1},x_{3},t\right)=\left\{\begin{array}{c} \begin{array}{cc} -x_{3}\ell_{f}^{\alpha -1}\overset{\alpha }{\underset{x_{1}}{D}}\left(\ell_{f}^{\alpha -1}\overset{\alpha }{\underset{x_{1}}{D}}\underset{⏜}{{\bar{u}}_{3}}\right)\;\;\;\;\;\; & \mathrm{for}\;\text{s-FEBB},\\ x_{3}\ell_{f}^{\alpha -1}\left[-\overset{\alpha }{\underset{x_{1}}{D}}\left(\ell_{f}^{\alpha -1}\overset{\alpha }{\underset{x_{1}}{D}}\underset{⏜}{{\bar{u}}_{3}}\right)+\overset{\alpha }{\underset{x_{1}}{D}}\underset{⏜}{{\overset{⋄}{\gamma }}_{13}}\right]\; & \mathrm{for}\;\text{s-FTB}, \end{array} \end{array}\right.$$

(A3) $${\overset{⋄}{\varepsilon }}_{13}={\overset{⋄}{\varepsilon }}_{13}\left(x_{1},t\right)=\left\{\begin{array}{c} \begin{array}{cc} 0\;\; & \mathrm{for}\;\text{s-FEBB},\\ \frac{1}{2}\underset{⏜}{{\overset{⋄}{\gamma }}_{13}}\; & \mathrm{for}\;\text{s-FTB}, \end{array} \end{array}\right.$$

and the corresponding stresses are

(A4) $$\sigma_{11}=\sigma_{11}\left(x_{1},x_{3},t\right)=\left\{\begin{array}{c} \begin{array}{cc} -x_{3}\ell_{f}^{\alpha -1}\overset{\alpha }{\underset{x_{1}}{D}}\left(\ell_{f}^{\alpha -1}\overset{\alpha }{\underset{x_{1}}{D}}\underset{⏜}{{\bar{u}}_{3}}\right)E\;\;\;\;\; & \mathrm{for}\;\text{s-FEBB},\\ x_{3}\ell_{f}^{\alpha -1}\left[-\overset{\alpha }{\underset{x_{1}}{D}}\left(\ell_{f}^{\alpha -1}\overset{\alpha }{\underset{x_{1}}{D}}\underset{⏜}{{\bar{u}}_{3}}\right)+\overset{\alpha }{\underset{x_{1}}{D}}\underset{⏜}{{\overset{⋄}{\gamma }}_{13}}\right]E\; & \mathrm{for}\;\text{s-FTB}, \end{array} \end{array}\right.$$

(A5) $$\sigma_{13}=\sigma_{13}\left(x_{1},t\right)=\left\{\begin{array}{c} \begin{array}{cc} 0\;\; & \mathrm{for}\;\text{s-FEBB},\\ G{\overset{⋄}{\varepsilon }}_{13}\; & \mathrm{for}\;\text{s-FTB}, \end{array} \end{array}\right.$$

where *E* is the Young’s modulus, $G=\frac{E}{2(1+\nu )}$ is the Kirchhoff modulus, and ν is Poisson’s ratio. As in the classical beam theories, the sectional stress resultants are introduced, namely, the bending moment $M_{2}$ and the shear force $V_{3}$ are expressed as

(A6) $$\underset{⏜}{M_{2}}=\int_{A}x_{3}\sigma_{11}\;dA\;\mathrm{and}\;\underset{⏜}{V_{3}}=\int_{A}\sigma_{13}\;dA.$$

The governing equations for transverse vibrations are derived from the Hamilton’s principle

(A7) $$\delta \Pi =\int_{t}\left(\delta U-\delta W-\delta K\right)\;dt=0,$$

where *U* is the internal energy, *W* is the external work, and *K* is the kinetic energy defined as follows:

(A8) $$\begin{array}{c} U=\frac{1}{2}\int_{L}\int_{A}\left(\overset{⋄}{\varepsilon_{11}}\sigma_{11}+2{\overset{⋄}{\varepsilon }}_{13}\sigma_{13}\right)\;dA\;dL=\frac{1}{2}\int_{L}\left[\underset{⏜}{M_{2}}\ell_{f}^{\alpha -1}\overset{\alpha }{\underset{x_{1}}{D}}\underset{⏜}{\Phi_{2}}+\left(\underset{⏜}{\Phi_{2}}+\ell_{f}^{\alpha -1}\overset{\alpha }{\underset{x_{1}}{D}}\underset{⏜}{{\bar{u}}_{3}}\right)\underset{⏜}{V_{3}}\right]\;dL, \end{array}$$

(A9) $$W=\int_{L}\underset{⏜}{p_{3}}\underset{⏜}{{\bar{u}}_{3}}\;dL,$$

(A10) $$K=\frac{1}{2}\int_{V}\rho {\dot{u}}^{2}\left(x_{1},t\right)\;dV=\left\{\begin{array}{c} \begin{array}{cc} \frac{1}{2}\int_{V}\rho {\underset{⏜}{{\dot{\bar{u}}}_{3}}}^{2}\;dV=\frac{1}{2}\int_{L}\rho A\underset{⏜}{{\dot{\bar{u}}}_{3}^{2}}\;dL\;\;\;\;\;\;\;\;\;\;\; & \mathrm{for}\;\text{s-FEBB},\\ \frac{1}{2}\int_{V}\rho \left(\underset{⏜}{{\dot{\bar{u}}}_{3}^{2}}+x_{3}^{2}\underset{⏜}{{\dot{\Phi }}_{2}^{2}}\right)\;dV=\frac{1}{2}\int_{L}\rho \left(A\underset{⏜}{{\dot{\bar{u}}}_{3}^{2}}+I\underset{⏜}{{\dot{\Phi }}_{2}^{2}}\right)\;dL\; & \mathrm{for}\;\text{s-FTB}, \end{array} \end{array}\right.$$

where $\dot{\left(\;\;\right)}=\frac{d(\;)}{dt}$. It should be emphasized that the kinetic energy *K* for the s-FTB model, compared to the s-FEBB model, includes an additional part $\frac{1}{2}\int_{V}\rho x_{3}^{2}\underset{⏜}{{\dot{\Phi }}_{2}^{2}}dV$ related to rotary inertia of cross section.

Next, substituting Equations (A8)–(A10) in Equation (A7) leads to

(A11) $$\delta \Pi =\begin{array}{c} \left\{\begin{array}{c} \begin{array}{cc} \int_{t}\int_{L}\left[\left(\underset{⏜}{M_{2}}\ell_{f}^{\alpha -1}\overset{\alpha }{\underset{x_{1}}{D}}\delta \underset{⏜}{\Phi_{2}}+\underset{⏜}{V_{3}}\delta \underset{⏜}{\Phi_{2}}\right)+\left(\ell_{f}^{\alpha -1}\underset{⏜}{V_{3}}\overset{\alpha }{\underset{x_{1}}{D}}\delta \underset{⏜}{{\bar{u}}_{3}}-\left(\underset{⏜}{p_{3}}-\rho A\underset{⏜}{{\ddot{u}}_{3}}\right)\delta \underset{⏜}{{\bar{u}}_{3}}\right)\right]dLdt=0\;\;\;\; & \mathrm{for}\;\text{s-FEBB},\\ \int_{t}\int_{L}\left[\left(\underset{⏜}{M_{2}}\ell_{f}^{\alpha -1}\overset{\alpha }{\underset{x_{1}}{D}}\delta \underset{⏜}{\Phi_{2}}+\left(\underset{⏜}{V_{3}}+\rho I\underset{⏜}{{\ddot{\Phi }}_{2}}\right)\delta \underset{⏜}{\Phi_{2}}\right)+\left(\ell_{f}^{\alpha -1}\underset{⏜}{V_{3}}\overset{\alpha }{\underset{x_{1}}{D}}\delta \underset{⏜}{{\bar{u}}_{3}}-\left(\underset{⏜}{p_{3}}-\rho A\underset{⏜}{{\ddot{u}}_{3}}\right)\delta \underset{⏜}{{\bar{u}}_{3}}\right)\right]dLdt=0\; & \mathrm{for}\;\text{s-FTB}, \end{array} \end{array}\right. \end{array}$$

and utilizing the fractional Euler–Lagrange equation [48,49], the relationships Equations (9) and (10) are obtained. However, introducing Equations (A4) and (A5) into Equation (A6) leads to Equation (7).

## Appendix B

The numerical representations of shear force Equation (11), bending moment Equation (7), and rotation Equation (3) at node $x_{1}^{i}$, utilizing the Equations (19) ÷ (27), are as follows:

(A12) $$V_{3}\left(x_{1}^{i}\right)=\left\{\begin{array}{c} \begin{array}{cc} -C_{i}EI\;\;\;\;\;\;\;\;\; & \mathrm{for}\;\text{s-FEBB},\\ \left\{\begin{array}{c} \left(-C_{i}+B_{i}\right)EI-\rho I\omega^{2}\left({\left(\ell_{f}^{\alpha -1}\right)}_{i}A_{i}-{\overset{⋄}{\gamma }}_{13}\left(x_{1}^{i}\right)\right)\\ kGA{\overset{⋄}{\gamma }}_{13}\left(x_{1}^{i}\right) \end{array}\right. & \mathrm{for}\;\text{s-FTB}, \end{array} \end{array}\right.$$

(A13) $$M_{2}\left(x_{1}^{i}\right)=\left\{\begin{array}{c} \begin{array}{cc} -{\left(\ell_{f}^{\alpha -1}\right)}_{i}B_{i}EI\;\;\; & \mathrm{for}\;\text{s-FEBB},\\ {\left(\ell_{f}^{\alpha -1}\right)}_{i}\left(-B_{i}+A_{i}\right)EI\; & \mathrm{for}\;\text{s-FTB}, \end{array} \end{array}\right.$$

(A14) $$\Phi_{2}\left(x_{1}^{i}\right)=\left\{\begin{array}{c} \begin{array}{cc} -{\left(\ell_{f}^{\alpha -1}\right)}_{i}A_{i}\;\;\;\; & \mathrm{for}\;\text{s-FEBB},\\ -{\left(\ell_{f}^{\alpha -1}\right)}_{i}A_{i}+{\overset{⋄}{\gamma }}_{13}\left(x_{1}^{i}\right)\; & \mathrm{for}\;\text{s-FTB}, \end{array} \end{array}\right.$$

It is also clear that, as in Equation (16), applying α=1 in Equations (18), (A12) ÷ (A14) causes a return for $N_{1}=0$ and $N_{2}=\frac{1}{2}$ (see also Table 1) to the classical central difference scheme ($A=\frac{1}{2}$, $B=C^{a}=C^{b}=0$ in Equation (26)):

(A15) $$\left\{\begin{array}{c} \begin{array}{cc} \frac{u_{i-2}-4u_{i-1}+6u_{i}-4u_{i+1}+u_{i+2}}{h^{4}}EI-\rho A\omega^{2}u_{i}=0\;\;\;\;\;\;\;\;\; & \mathrm{for}\;\mathrm{CEBB},\\ \left\{\begin{array}{c} \begin{array}{cc} \; & \frac{u_{i-2}-4u_{i-1}+6u_{i}-4u_{i+1}+u_{i+2}}{h^{4}}EI-\frac{-\gamma_{i-3/2}+3\gamma_{i-1/2}-3\gamma_{i+1/2}+\gamma_{i+3/2}}{h^{3}}EI+\\ \; & \;\;\;\;+\rho I\omega^{2}\frac{u_{i-1}-2u_{i}+u_{i+1}}{h^{2}}-\rho A\omega^{2}u_{i}-\rho I\omega^{2}\frac{-\gamma_{i-1/2}+\gamma_{i+1/2}}{h}=0\\ \; & \frac{-u_{i-3/2}+3u_{i-1/2}-3u_{i+1/2}+u_{i+3/2}}{h^{3}}EI-\frac{\gamma_{i-1}-2\gamma_{i}+\gamma_{i+1}}{h^{2}}EI+\\ \; & \;\;\;\;+\rho I\omega^{2}\left(\frac{-u_{i-1/2}+u_{i+1/2}}{h}-\gamma_{i}\right)+kGA\gamma_{i}=0 \end{array} \end{array}\right.\; & \mathrm{for}\;\mathrm{CTB}, \end{array} \end{array}\right.$$

(A16) $$V_{i}=\left\{\begin{array}{c} \begin{array}{cc} -\frac{-u_{i-3/2}+3u_{i-1/2}-3u_{i+1/2}+u_{i+3/2}}{h^{3}}EI\;\;\;\;\;\;\;\;\;\;\; & \mathrm{for}\;\mathrm{CEBB},\\ \begin{array}{cc} (- & \frac{-u_{i-3/2}+3u_{i-1/2}-3u_{i+1/2}+u_{i+3/2}}{h^{3}}+\frac{\gamma_{i-1}-2\gamma_{i}+\gamma_{i+1}}{h^{2}})EI+\\ & \;-\rho I\omega^{2}\left(\frac{-u_{i-1/2}+u_{i+1/2}}{h}-\gamma_{i})\right), \end{array}\; & \mathrm{for}\;\mathrm{CTB} \end{array} \end{array}\right.$$

(A17) $$M_{i}=\left\{\begin{array}{c} \begin{array}{cc} -\frac{u_{i-1}-2u_{i}+u_{i+1}}{h^{2}}EI\;\;\;\;\;\;\;\;\;\;\;\; & \mathrm{for}\;\mathrm{CEBB},\\ \left(-\frac{u_{i-1}-2u_{i}+u_{i+1}}{h^{2}}+\frac{-\gamma_{i-1/2}+\gamma_{i+1/2}}{h}\right)EI\;\; & \mathrm{for}\;\mathrm{CTB}, \end{array} \end{array}\right.$$

and

(A18) $$\Phi_{i}=\left\{\begin{array}{c} \begin{array}{cc} -\frac{-u_{i-1/2}+u_{i+1/2}}{h}\;\;\;\; & \mathrm{for}\;\mathrm{CEBB},\\ -\frac{-u_{i-1/2}+u_{i+1/2}}{h}+\gamma_{i}\; & \mathrm{for}\;\mathrm{CTB}, \end{array} \end{array}\right.$$

where $\Phi_{i}=\Phi_{2}\left(x_{1}^{i}\right)$, $M_{i}=M_{2}\left(x_{1}^{i}\right)$, $V_{i}=V_{3}\left(x_{1}^{i}\right)$, $p_{i}=p_{3}\left(x_{1}^{i}\right)$, $\gamma_{i}=\gamma_{13}\left(x_{1}^{i}\right)$ and $u_{i}={\bar{u}}_{3}\left(x_{1}^{i}\right)$.
